# Diseases of the Enamel

**Published:** 1875-02

**Authors:** Henry S. Chase

**Affiliations:** St. Louis, Mo.


### ARTICLE IV.

### *Diseases of the Enamel.*

BY HENRY S. CHASE, M. D., ST. LOUIS, MO.

Notwithstanding the compact structure of this substance it is in early life subject to inflammations and malnutrition; and at a later period, though not manifesting the same signs of inflammation that is exhibited in soft tissues, yet it undoubtedly goes through some of the movements which result in inflammation and death of the tissue.

During the development of the permanent teeth and before eruption, or even after eruption, all those portions of the dental crowns which are still covered by the gum, or within their respective sockets are peculiarly liable to be injuriously affected by eruptive disease accompanied by much fever. Scarlet Fever, Measles, and Small Pox leave their impress upon the enamel with great certainty. The enamel organ participates in the dermoid inflammation, and the enamel prisms cannot by any possibility escape. The delicate sheath which envelops every prism, being of the same histological structure as the enamel membrane itself is easily affected, and every prism may undergo inflammation. These delicate sheaths constitute the *basal* structure of the enamel. It is into these cells the fluid phosphate of lime is invested and becomes crystalized. Upon the severity and extent of the inflammatory process depends the appearance and physical condition of the enamel after the tooth is erupted.

Let us keep in mind that at this period cell life is in its greater activity, every thing is in motion ; work is going on incessantly in every organ, and every tissue. *Building* is in progress ; but if the workmen are sick, the process is arrested. When there is general disease the appetite fails ; digestion suffers ; food is not made into pabulum, protoplasm is deficient, and there are no *materials* for building, even if the builders were able to work. So *here* if nutrition is arrested then absorption will predominate, and a line of depression show around the tooth after its eruption. This will depend on the length of time during which the development of the organ was arrested, and whether wholly, or partially so. Wool of sheep ; hair, and nails show the marks of defective, or arrested nutrition, caused either by sickness or want of food.

I have to day, July 15th, the depression or groove running across each thumb nail caused by a fortnight's sickness in March. The depressions or shallow grooves so often observed running across the incisor and other teeth, are the result of arrested development. The white, soft, chalk like spots on the enamel are caused by inflammation of the enamel cells, or sheaths of enamel columns, while yet the phosphate of lime was uncrystalized. Stasis resulted, and the lime in that chalky enamel to-day is not crystalized ; consequently there is no firmness or density to the tissue.

That enamel which appears seedy, or granular and still hard, was produced by inflammation and shrivelling of the enamel membrane, after the enamel cells had crystallized their contents.

In healthy and naturally developed enamel there is a circulation of the plasma of the blood through every enamel cell membrane, not by vessels, of course, but through osmotic action, such as takes place in every part of the body, at a greater or less distance from arterial vessels. To deny this is absurd.

In every portion of the body there is a territory outside of any nutritient canals, which is constantly importing and exporting products. The activity of these motions depends much on the age of the individual, for it is well known that as age increases, nearer and nearer a static condition is reached, which at last may become perfect in some of the hard tissues, and especially in the enamel.

It is important to keep this in mind when we are making observations upon the teeth after eruption.

Every experienced observer knows that all of the hard tissues of the teeth are in a state of mobility before the fifteenth year, and I shall say, for a long time afterwards. The enamel has not upon its exterior that vitreous appearance that it has later in life. This may be owing to the more perfect crystalizations of the lime salts at a later period. As we extend our investigations deeper into the enamel substance towards the dentine we find less and less compactness, for that portion lying next to the dentine hardens later than the exterior portion. The exterior is always in advance of the interior; the same remarks will apply to the dentine, but in the later nutritive changes take place to a greater extent, and more rapidly than in the enamel, just as might be expected, for it is a softer tissue. A large number of observers have demonstrated that absorption often takes place in the dentine, and a much smaller number have demonstrated that it does, in rarer cases, in the enamel itself. We have seen that inflammation of the enamel membrane before eruption causes necrosis of that tissue, and now we wish to see how that pathological result may be observed to take place in the already erupted tooth. Let me tell you what I have hundreds of times seen and then we will try to arrive at a theory which will explain the phenomena. I am looking into a fifteen year old mouth and I see the anterior surface of a right under first molar. It is smooth and vitreous in appearance, and has no abrasion or crack on its surface, but there is a whitish spot about the centre of its surface. It is "off color." It has lost its polish; it cuts like rock salt; as I penetrate further it cuts easier until I arrive at the dentine, which is as hard as we find it at eight years after eruption.

I see the same tooth in the mouth of a person aged twenty. The spot is *brown* instead of white, it has a vitreous surface, smooth and without sign of disintegration. It cuts hard at the surface but immediately the thickness of tissue paper is cut through, the enamel is found friable, chalky to the feeling, and when the dentine is reached the latter is found more or less *softer than natural*, apparently decalcified, and a circumscribed cavity is formed, bounded by harder tissue.

I see the same tooth and surface in the mouth of a person aged twenty-five. The spot is black, vitreous surface; no disintegration. The blackness penetrates the enamel, shading off into dark brown until it reaches the dentine, where it still shades off into lighter brown, until the natural color of healthy dentine is reached. This black spot vitreous in hardness and polish, cuts at first like very thin feldspar but grows less dense, not seeming porous, as I go in, then becomes granular, and when the dentine is reached it cuts something like hard wood across the ends of its fiber, shading off into normally hard adult dentine. The previous condition of this tooth was that of pressure, caused by one now extracted. I have observed so many cases of this kind that I can attribute the pathology to nothing but necrosis of the enamel, caused by pressure, followed by stasis of circulation in enamel cells; a similar condition being caused by inflammation extending to dentine tubes, and decalcification of dentine previous to necrosis of the latter substance.

Sponstaneous abrasion, or denudation can only be fully explained by a *theory* which covers the ground which I have gone over, namely that vital action plays an important part in all pathological conditions of the dentine and enamel.—*Missouri Dental Journal.*

